# Prediction of Damage Distribution in Gas Cylinder Stages Based on Semi-Supervised and Transfer Learning Algorithms

**DOI:** 10.3390/s26134014

**Published:** 2026-06-24

**Authors:** Xiangdong Ma, Zhigang Gao, Wenli Dong, Shen He, Zhongyuan Xu, Xiao Wu, Wei Zheng, Jiongming Wen, Yonghua Yu

**Affiliations:** Special Equipment Safety Supervision Inspection Institute of Jiangsu Province, Nanjing 210036, China

**Keywords:** fiber-reinforced composite cylinder, prediction of damage distribution, semi-supervised, transfer learning algorithms, mean-teacher network structure

## Abstract

Currently, clustering algorithms are mainly used to classify fiber-reinforced composite cylinder damage. However, the number of clustering categories is heavily influenced by the evaluation criteria, and the real damage type categorization cannot be determined. Therefore, we propose a semi-supervised algorithm that obtains higher damage classification information with a small number of labels. Specifically, we first performed a phased fiber-reinforced composite cylinder pressurization experiment and collected damage signals through acoustic emission (AE) hits. We analyzed the damage types of the collected burst-type acoustic emission hits (each hit corresponds to a single waveform captured when the hit’s amplitude exceeds the preset threshold) and marked a small number of these hits. Then, we constructed a mean-teacher semi-supervised network structure based on transfer learning, achieving a classification accuracy of 85.92%. Compared to traditional supervised learning and clustering algorithms, the accuracy improved by nearly 30%.

## 1. Introduction

At present, hydrogen is becoming a promising future energy medium in various industries. For mobile applications, it is usually stored in gaseous form in high-pressure composite external packaging pressure vessels [1]. As lightweight and high-strength containers, mobile pressurized gas containers composed of multiple materials, such as fiber-wound composite (FRP) gas cylinders, have the advantages of light weight, high strength, corrosion resistance, and good fatigue performance [2]; they are key components in many industries and applications [3]. In the automotive industry, they are used in hydraulic systems, air compressors, and braking systems [1]. They are also of great significance in aeronautics because they play key functions in various aircraft systems, including hydraulic power units and fuel storage [4].

As pressure-bearing equipment, the FRP gas cylinder generally has flammable, explosive, toxic, and strongly corrosive properties for its containment medium. During its service period, due to loads and other external factors, internal damage will continuously accumulate [5], and failure modes of composite layers such as fiber breakage, matrix cracking, and interlaminar delamination [6] will eventually lead to the overall failure of the gas cylinder. This will pose safety risks to personnel involved.

Acoustic emission technology is a passive, dynamic, non-destructive testing method that can effectively detect the formation and development of internal structural defects in materials and evaluate the safety of the overall structure. It can capture the acoustic wave signals generated within the material, thereby enabling accurate evaluation of the internal defects [5]. At present, many scholars at home and abroad have carried out damage assessment research on composite pressure vessels based on acoustic emission [7,8,9,10,11]. Therefore, it is extremely important to simulate the load conditions of composite pressure vessels in service in experiments and study the damage mechanisms, damage evolution, and damage assessment of composite pressure vessels through acoustic emission detection technology [12].

In 2021, Hao et al. [13] used the finite element analysis method to predict the burst pressure and fatigue life of gas cylinders, analyzing the stress distribution inside the gas cylinders. In 2022, Sikdar et al. [14] proposed a deep learning structure based on a convolutional neural network to automatically extract discrete damage features from scale map images. In 2022, Xu et al. [15] used acoustic emission technology to monitor the performance degradation process of glass fiber/epoxy composite laminates and constructed a prediction model of acoustic emission signals. In 2022, Harizi et al. [16] combined monotonic tensile and step-by-step tensile experimental data of glass fiber-reinforced polymer composites to evaluate the acoustic characteristics of each damage mechanism and identify them according to the applied load level. In 2024, Wei et al. [17] analyzed AE hits from gas cylinder damage and compared the parameter values and change trends synchronously through the Mel frequency cepstrum coefficient (MFCC) feature extraction method. The test results show that the distribution of the Mel frequency cepstrum coefficients of different damage types shows obvious regularity. Gao et al. proposed a multi-scale physical neural network to overcome structural limitations in multiaxial fatigue life prediction, offering a new perspective for damage modeling and potential improvements for acoustic emission-based classification [18]. In this study, the classification unit is defined as an individual AE hit. Unlike continuous AE hits, the damage-induced signals in composite pressure vessels are predominantly burst-type, which exhibit distinct time–frequency characteristics.

In conclusion, many domestic and foreign experts and scholars have analyzed and studied the damage detection problem of FRP gas cylinders, but large computational volumes and complex and cumbersome computational processes remain significant challenges. Based on this, we propose a mean-teacher semi-supervised network structure algorithm combined with transfer learning for acoustic emission (AE) damage identification under limited-label conditions. In practical AE applications, obtaining reliable labels requires expert knowledge and detailed interpretation of signal characteristics, which is time-consuming and labor-intensive. Therefore, reducing the dependence on large-scale labeled datasets is a key challenge in AE-based damage classification. To address this issue, the proposed method leverages a small amount of labeled data together with a large quantity of unlabeled data through a semi-supervised learning framework, enabling effective damage classification while significantly reducing manual annotation cost. Compared to traditional supervised or clustering-based approaches, this framework is more consistent with real engineering scenarios, where unlabeled AE data are abundant but labeled data are scarce.

This study proposed a semi-supervised learning algorithm based on mean-teacher and transfer learning, aiming to achieve high classification accuracy with a small amount of labeled data. A phased pressure damage experiment was conducted on FRP gas cylinders, where a hydrostatic test generated 118,880 AE hits. The time-domain and frequency-domain characteristic parameters of the AE hits from different channels were analyzed. A total of 118,880 burst-type AE hits were recorded across nine stepwise pressurization stages. Among the hits from the first seven stages (29,825 hits), only 1590 hits, approximately 5.3% of the total, were manually labeled due to the high cost of expert annotation. These sparse labels, together with the abundant unlabeled hits from the same stages, were used to train a semi-supervised mean-teacher network. An independent test set of 1470 labeled hits from Stage 8 was reserved for performance evaluation. The trained model was then applied to predict the damage distribution of Stage 9 (23,426 hits) without any labels. Based on the data analysis from the first seven stages of the experiment, damage classification prediction was performed for the AE hits from the eighth and ninth stages. The results revealed that the main damage types in the gas cylinders were delamination, fiber fracture, matrix cracking, and fiber pullout.

## 2. Experiment

**Figure 1:**
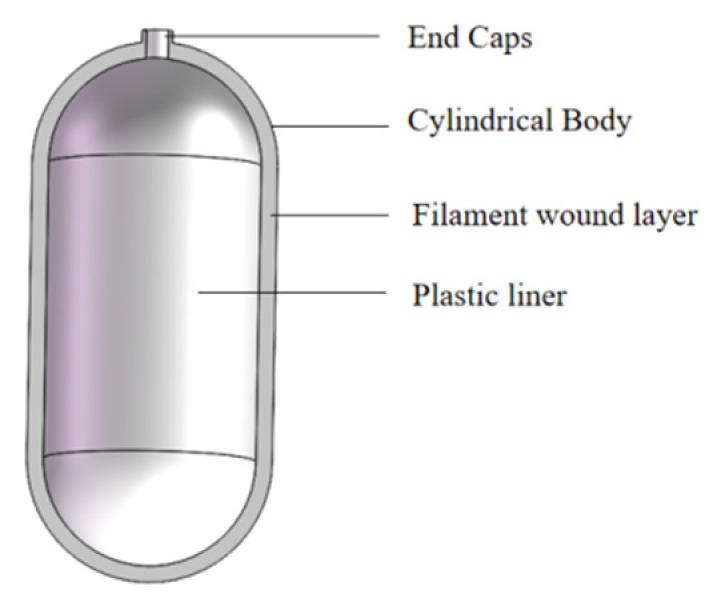
General structural schematic diagram of fiber-wrapped plastic inner cylinder.

The general structure of the FRP gas cylinder used in the experiment is shown in Figure 1.

The FRP gas cylinder in this experiment is manufactured by Tianjin Anyida Composite Gas Cylinder Co., Ltd., Tianjin, China. The container model is FYSP-G-18-A, the product standard code is T/CATSI 02005-2019 [19] and the full name of the container is liquefied petroleum gas high-density polyethylene inner liner glass fiber fully wound gas cylinder. A hydrostatic test of 3.2 MPa is carried out before leaving the factory. This gas cylinder uses epoxy resin, uses E-CR-type glass fiber, and has a high-density polyethylene inner liner. The main parameters are shown in Table 1 and Table 2.

**Figure 2:**
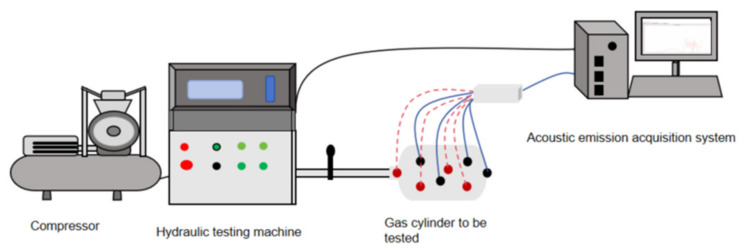
Schematic diagram of acoustic emission monitoring gas cylinder water pressure test platform.

The experiment uses a SUPERTOK hydrostatic pressure testing machine (Kunshan Leiken Hydraulic System Technology Co., Ltd., Kunshan, China) and an emission acquisition system composed of a PCI-Express AE acquisition system (model: Express-8, Physical Acoustics Corporation, a MISTRAS Group company, Princeton Junction, NJ, USA), 2/4/6 preamplifiers with a gain of 40 dB, and WD broadband AE sensors (model WD, frequency range 100–1000 kHz, Physical Acoustics Corporation, a MISTRAS Group company, Princeton Junction, NJ, USA) to construct a high- and low-pressure hydrostatic test and establish an acoustic emission detection FRP gas cylinder hydrostatic test monitoring platform to conduct a graded loading hydrostatic test. The hydrostatic pressure testing machine in the experimental platform can realize low-pressure 0–5 MPa loading and high-pressure 0–100 MPa loading. During the experimental loading, the hydrostatic test system can introduce the real-time pressure value of the pressure transmitter into the acoustic emission acquisition system. The connection diagram of this experimental platform is shown in Figure 2.

**Figure 3:**
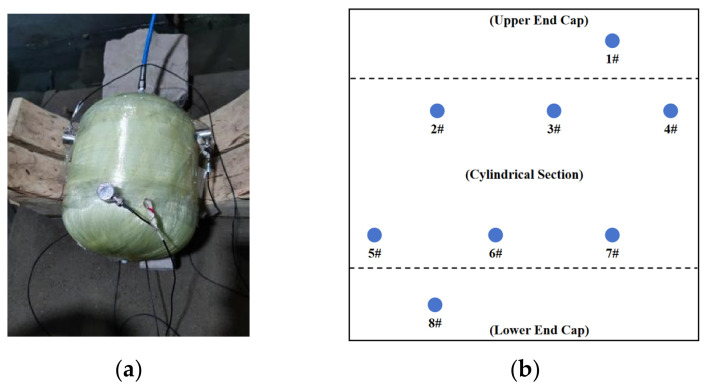
Experimental schematic diagram: (**a**) water pressure test diagram of gas cylinder (left), (**b**) 2D diagram of sensor layout (right).

The experimental scheme is shown in Figure 3. Sensor arrangement: A total of eight WD broadband sensors are used in the acoustic emission detection experiment arrangement. Among them, sensors 1# and 8# are arranged at the upper and lower heads, respectively. Sensors 2#, 3#, and 4# are evenly distributed along the upper transition section of the gas cylinder. Sensors 5#, 6#, and 7# are evenly distributed along the lower transition section, but are circumferentially staggered relative to sensors 2#, 3#, and 4#, and the AE sensors were arranged based on a zonal monitoring strategy, where different structural regions of the pressure vessel were covered by specific sensor groups. During the experiment, the gas cylinder is placed horizontally on the experimental platform frame. A coupling agent is applied to the surface between the sensor and the winding layer of the gas cylinder to achieve better coupling conditions and conduct coupling standard verification of coupling performance.

AE data acquisition was performed using the Express-8 PCI-Express AE acquisition system. To ensure the validity of the experimental acquisition data, background noise should be measured before the experiment. According to the environmental noise measurement results before the experiment, the threshold is set to 40 dB for this experiment. The acoustic emission system parameter settings are shown in Table 3.

Type of AE data acquired: During the hydrostatic step-loading test, all recorded AE hits were of the burst type, triggered by sudden energy release from matrix cracking, fiber breakage, debonding, or fiber pullout. No continuous AE hits were included in the analysis. Each recorded hit was treated as an independent classification sample.

**Figure 4:**
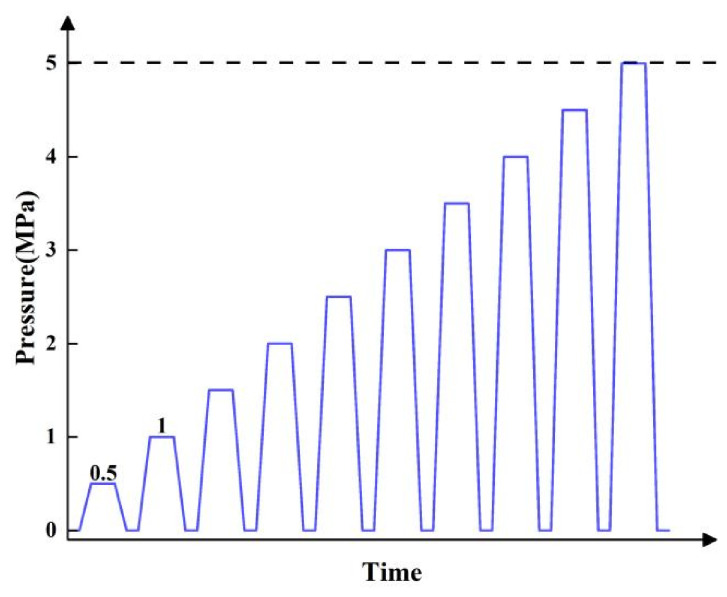
Ladder pressurization program diagram.

Experimental loading scheme:

(a) Install the acoustic emission sensor as shown in Figure 3a and set the parameters of the acoustic emission detection system. To ensure the airtightness of the device during the experiment, an airtightness pressurization experiment is carried out before the experiment to test the airtightness of the device (in this experiment, pressurization to 0.5 MPa, pressure holding for 60 s, and pressure relief are selected).

(b) To ensure both the validity of the data and that there are no various types of noise for AE monitoring during the experiment, perform background noise calibration before the experiment is loaded. The experiment follows a cycle of pressurizing by 0.5 MPa each time, holding the pressure for 4 min, and then depressurizing.

(c) The hydrostatic test program scheme is as follows: airtightness of the experimental device, background noise calibration, and step pressurization. The step pressurization curve is shown in Figure 4.

## 3. Methods

**Figure 5:**
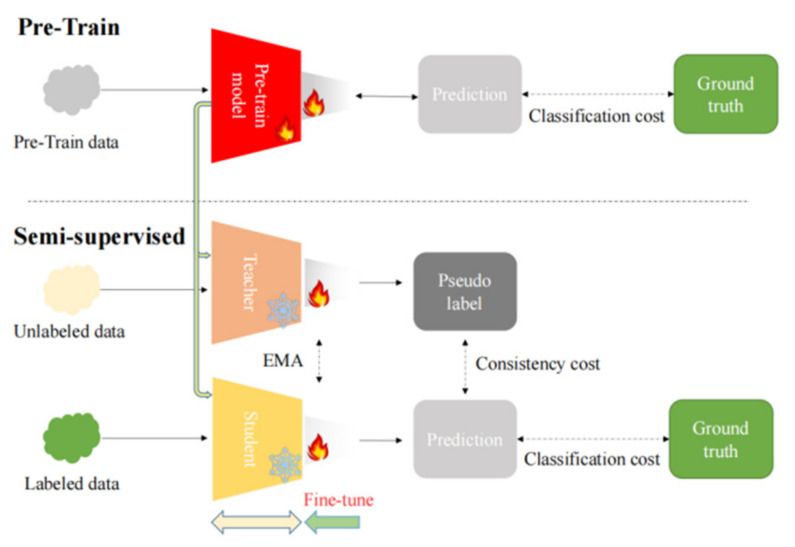
Method flow chart.

The proposed method follows a semi-supervised mean-teacher framework combined with transfer learning, as illustrated in Figure 5. For each burst-type AE hit, we extracted a time-domain waveform and converted it into a time–frequency representation using CWT. The CWT scalogram of each hit (size 224 × 224 × 3) serves as the input to the mean-teacher network. No feature aggregation across multiple hits was performed, so the classification unit remains the individual AE hit. Below, we detail the conversion procedure, network structure, and training process.

Conversion from AE hits to image representations. Each burst-type AE hit is a one-dimensional time-domain waveform captured when the hit amplitude exceeds the preset threshold. The sampling rate is 1 MHz, and the waveform length is determined by the PDT and HDT settings, resulting in approximately 1000 sampling points per hit. To extract discriminative damage-related features, we apply CWT to each waveform. CWT provides a time–frequency scalogram that reveals how frequency components evolve over time, which is particularly suitable for burst-type AE hits with transient characteristics. The CWT of a signal is defined as follows:

(1) $$W\left(a,b\right)=\int_{-\infty }^{\infty }s\left(t\right)\frac{1}{\sqrt{a}}\psi^{*}\left(\frac{t-b}{a}\right)dt$$

where a is the scale parameter, b is the translation parameter, and ψ(t) is the mother wavelet. We choose the Morlet wavelet (‘morl’) as the mother wavelet because it provides a good balance between time and frequency localization for AE hit waveforms. The scale parameter is set to 256, producing a scalogram of size 256 × (number of time samples). This scalogram is a grayscale image. To match the input format required by ResNet50, we convert the grayscale scalogram into a pseudo-RGB image by duplicating the same matrix across three channels. The resulting image is then resized to 224 × 224 × 3 using bilinear interpolation. Finally, we normalize the pixel values using the mean and standard deviation of the ImageNet dataset (mean = [0.485, 0.456, 0.406], std = [0.229, 0.224, 0.225]) to align with the pre-trained model’s expectations.

Here, ResNet50 is used as the backbone network. ResNet50 is a 50-layer deep residual network composed of an initial convolution layer and max-pooling layer, followed by four residual stages with bottleneck blocks, and a final fully connected classification layer. The residual connections help alleviate the degradation problem in deep networks and improve feature extraction capability. More architectural details of ResNet can be found in He et al. [20].

Network structure and training. Both the student model $f_{\theta }$ and the teacher model $f_{\theta^{\prime }}$ share a ResNet50 backbone pre-trained on ImageNet. Let $D_{l}={\left\{\left(x_{i},y_{i}\right)\right\}}_{i=1}^{N_{l}}$ denote the labeled dataset, where $x_{i}$ is the CWT scalogram image of an AE hit and $y_{i}\in \{1,2,3,4\}$ corresponds to four damage types: matrix cracking, fiber/matrix debonding, fiber fracture, and fiber pullout. Let $D_{u}={\left\{\left(x_{j}\right)\right\}}_{j=1}^{N_{u}}$ N_u_ be the unlabeled dataset, with $N_{l}\ll N_{u}$. The teacher parameters $\theta^{\prime }$ are updated via exponential moving average (EMA) of the student parameters $\theta :\theta_{t}^{\prime }=\alpha \theta_{t-1}^{\prime }+\left(1-\alpha \right)\theta_{t}$, where α = 0.999 is the decay coefficient. The total loss is $L_{total}=L_{cls}+\lambda L_{cons}$ with λ = 0.1. The classification loss $L_{cls}$ is binary cross-entropy (BCE) computed on labeled data:

(2) $$L_{cls}=-\frac{1}{\left|D_{l}\right|}\sum_{\left(x_{i},y_{i}\right)\in D_{l}}\sum_{c=1}^{4}y_{i,c}log\left(f_{\theta }{\left(x_{i}\right)}_{c}\right)$$

The consistency loss $\lambda L_{cons}$ is the mean squared error (MSE) between student and teacher predictions on unlabeled data:

(3) $$L_{cons}=\frac{1}{\left|D_{u}\right|}\sum_{x\in D_{u}}∥f_{\theta }\left(x\right)-f_{\theta^{\prime }}\left(x\right){∥}^{2}$$

Training uses the SGD optimizer with a batch size of 16 for 100 epochs on an NVIDIA GeForce RTX 3090 GPU (NVIDIA Corporation, Santa Clara, CA, USA). After training, the teacher model predicts damage types for the unlabeled hits, and the results are aggregated into damage distribution pie charts.

The evaluation matrix is composed of accuracy, precision, recall, and F1. The definitions of each item are as follows:

Accuracy: The percentage of correctly predicted results in the total sample. Its expression is as follows (4):

(4) $$Accuracy=\frac{TP+TN}{TP+TN+FP+FN}$$

Precision: Among the correctly predicted samples, the percentage of actually correct samples. Its expression is as follows (5):

(5) $$Precision=\frac{TP}{TP+FP}$$

Recall: Among all correct samples, the percentage of actually correctly predicted ones. Its expression is as follows (6):

(6) $$Recall=\frac{TP}{TP+FN}$$

F1: The F1-score is an index used in statistics to measure the accuracy of a binary classification model. Its expression is as follows (7):

(7) $$F1\;Score=\frac{2\times (Precision\times Recall)}{Precision+Recall}$$

where TP (true positive) is a true positive example, TN (true negative) is a true negative example, FP (false positive) is a false positive example, and FN (false negative) is a false negative example.

In this way, the student model can approach or even reach the performance of the teacher model at a lower computational cost.

## 4. Results and Analysis

### 4.1. Acoustic Emission Hit Analysis and Damage Classification

In this study, a threshold of 40 dB was applied during AE data acquisition to suppress low-amplitude signals. As a result, most reflected and attenuated signals, which typically exhibit lower amplitudes after propagation, are effectively filtered out. This helps reduce the influence of secondary wave components and improves the reliability of the recorded AE hits.

Furthermore, the AE sensors were arranged based on a zonal localization strategy. Under this configuration, AE hits are primarily analyzed within their corresponding monitoring regions, which reduces the variability introduced by long-distance propagation paths across different sensors.

Although a single physical AE event may still generate multiple hits due to wave propagation effects, the combined use of amplitude thresholding and zonal sensor arrangement mitigates the impact of propagation-induced distortions to a certain extent. Therefore, the dominant frequency-related characteristics associated with damage mechanisms can still be effectively captured at the hit level.

**Figure 6:**
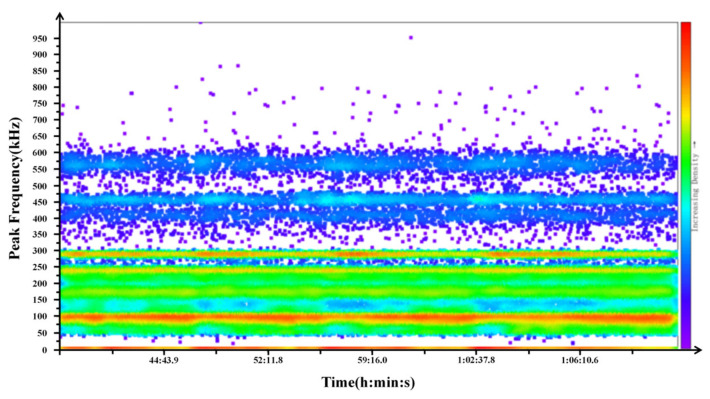
Time-peak frequency density plot of AE hits.

To represent the AE hits obtained from the experiment more intuitively, this paper uses a visualized point density diagram to analyze the damage types generated by the FRP gas cylinder during the experiment. Each point in Figure 6 corresponds to one AE hit and its peak frequency and occurrence time. The redder the color, the more hits share that peak frequency. The high-density regions indicate the frequency ranges where damage-related hits are concentrated. The point density diagram drawn according to the peak frequency–time relationship is shown in Figure 6. The construction details are as follows: the peak frequency of each AE hit is directly obtained from the AE acquisition system as a recorded hit parameter, and the occurrence time is also recorded by the acquisition system. Importantly, this plot is used only for visualizing the overall distribution of AE hits and guiding manual labeling; the actual input to the proposed mean-teacher network is the CWT scalogram of each individual AE hit, as described in Section 3. Specifically, the peak frequency recorded by the AE acquisition system was used to assign initial damage labels according to the predefined frequency ranges, while the CWT scalogram of each AE hit waveform was used as the image input to the Mean-Teacher network.

It can be seen from Figure 6 that the acoustic emission signals obtained from the experiment are mainly concentrated in the range of 50–300 kHz. There is also a large number of AE hits around 450 kHz and 560 kHz. According to the experimental results of Liao et al. [21] from Zhejiang University, the obtained signals are divided into four categories, below 175 kHz, 175–300 kHz, 400–500 kHz, and 500–600 kHz, corresponding to four damage modes of matrix cracking, fiber/matrix debonding, fiber fracture, and fiber pullout, respectively. It should be noted that the frequency bands are first identified based on the experimental data distribution in Figure 6 and then correlated with damage modes according to the existing literature, showing good consistency with previous studies.

The labeling of AE hits is based on frequency-domain characteristics rather than arbitrary assignment. As shown in Figure 6, the signals exhibit clear banded distributions, mainly concentrated in 50–300 kHz, with additional high-frequency components around 450 kHz and 560 kHz. According to Liao et al. [21], the AE signals can be divided into four frequency bands corresponding to different damage modes: below 175 kHz (matrix cracking), 175–300 kHz (fiber/matrix debonding), 400–500 kHz (fiber fracture), and 500–600 kHz (fiber pullout). In this study, labeling is performed based on these frequency criteria, ensuring physical interpretability and consistency. This frequency-domain-based damage characterization is also consistent with previous studies showing that multivariable analysis and wavelet-transform-based methods can effectively support the clustering and identification of acoustic emission signals in polymer-based composite materials [22].

### 4.2. Training and Testing Results for Damage Prediction

The labeled data and unlabeled data from Stages 1 to 7 were used for training, and the labeled data of Stage 8 were used for testing, and the damage of Stage 9 was predicted. Table 4 shows the total number of acoustic emission hits generated and the number of manually labeled hits in each stage.

To evaluate the advantage of semi-supervised learning under limited labeled data, we compared the proposed method against four baselines: K-means clustering [23], supervised learning [24], supervised learning with transfer learning, and semi-supervised learning alone. All methods were tested on the same Stage 8 dataset using accuracy, precision, recall, and F1-score, with labeled training data consisting of only 1590 manually annotated hits from Stages 1–7, which is approximately 5.3% of the total hits in those stages. The remaining unlabeled hits from Stages 1–7 were also used during training for the semi-supervised methods, while the supervised baselines used only the 1590 labeled hits. On the test set, supervised learning, transfer learning, semi-supervised learning, and semi-supervised learning combined with transfer learning are compared. The specific comparison results for different methods tested on the data from Stage 8 are presented in Table 5. As shown in Table 5, the transfer-learning-based models achieve consistently better mean performance than the corresponding models trained from scratch. In the semi-supervised setting, the accuracy increases from 85.03 ± 0.41 to 85.92 ± 0.82, and the F1-score increases from 85.07 ± 0.40 to 86.01 ± 0.25. Similar improvements can also be observed in the supervised setting. Overall, these results demonstrate that pretrained initialization is effective for improving the performance of the proposed model.

According to the obtained AE hits and classification methods, five experiments were conducted: K-means, fully supervised learning, fully supervised learning plus transfer learning, semi-supervised learning, and semi-supervised learning plus transfer learning. The evaluation indices are shown in Table 5 According to the table, we can clearly see that the experimental accuracy obtained by only using the K-means method is very low, only 25.37; the experimental accuracy obtained by only using the supervised learning method is not high either, only 47.62; and the experimental accuracy obtained by using the semi-supervised learning method is nearly 30% higher than that obtained by only using the fully supervised learning method. After adding transfer learning, the accuracy of both fully supervised learning and semi-supervised learning was improved to a certain extent.

It should be noted that K-means is an unsupervised clustering method, whereas our proposed method belongs to a semi-supervised classification framework that leverages label information. Since the two differ in learning paradigm and objective function, the direct accuracy comparison in Table 5 is provided only as a reference to illustrate the benefit of incorporating even a small number of labels, rather than as an equivalent methodological comparison.

The goal of the K-means clustering algorithm is to minimize the sum of the squares of the distances between all vectors in the data set and their centers [25]. The silhouette coefficient of the clustering result is the average of all samples. A silhouette coefficient close to 1 indicates that the clustering of samples is reasonable.

**Figure 7:**
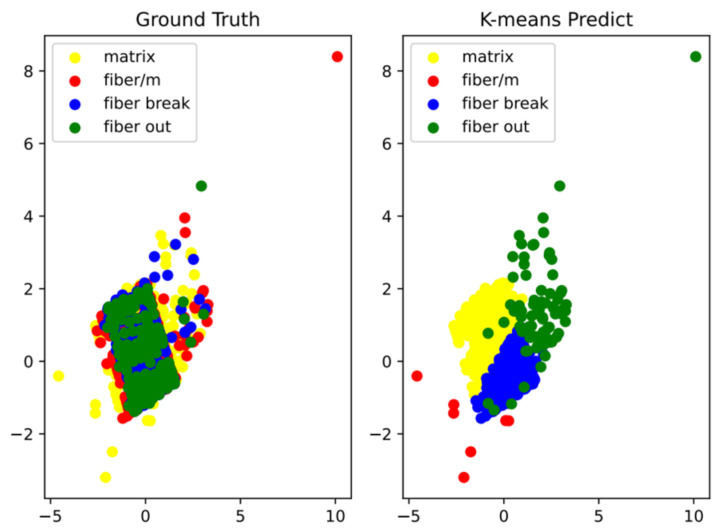
Comparison diagram of K-means clustering results and real labels.

It can be seen from Figure 7 that there is a huge difference between the K-means clustering results and the real labels, indicating that the K-means clustering results are not accurate and do not reflect the real damage situation of the gas cylinder.

### 4.3. Loss Comparison and Model Performance Evaluation

**Figure 8:**
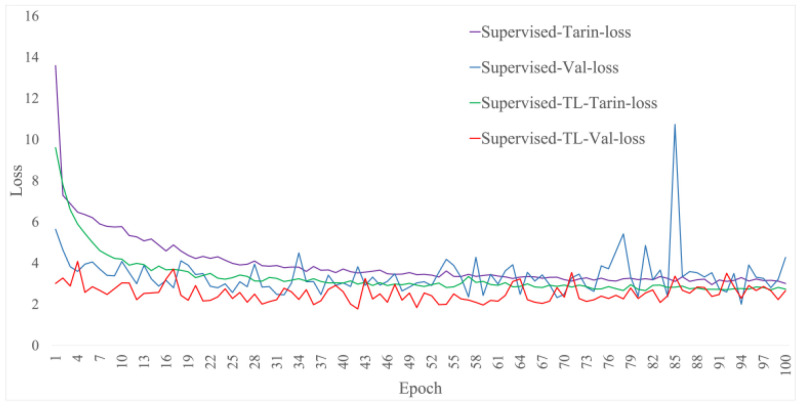
Comparison diagram of loss between supervised learning and transfer learning.

**Figure 9:**
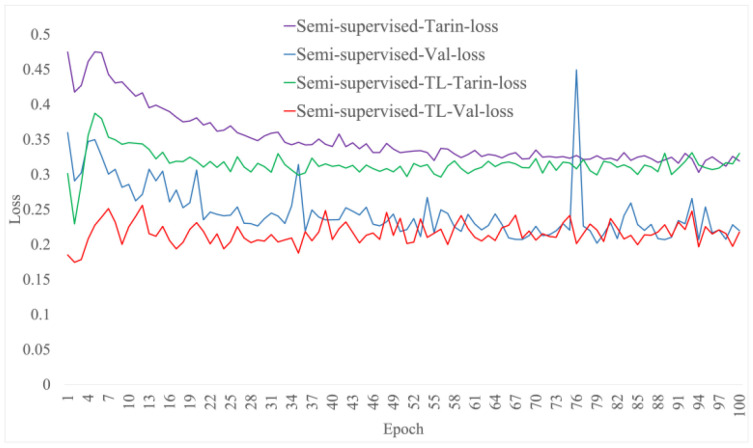
Comparison diagram of loss of semi-supervised learning and transfer learning.

The training and validation losses shown in Figure 8 and Figure 9 are both calculated using splits from the Stage 1–7 dataset, whereas the labeled data from Stage 8 are used only for the final performance evaluation. It can be seen from Figure 8 that transfer learning has a lower loss on both the training set and the validation set. This indicates that the features extracted by the pre-trained backbone network are more robust. It can also be seen from the figure that in the later stage of training, the transfer learning method is more stable on the validation set. While the network without using pre-trained parameters shows overfitting in the later stage of training, the loss on the validation set fluctuates greatly.

It can be seen from Figure 9 that semi-supervised learning combined with the transfer learning method has better fitting distribution ability, and the loss using the transfer learning method is more stable on the validation set. Comparing Figure 8 and Figure 9, it can be seen that the semi-supervised learning plus transfer learning method can effectively improve the recognition accuracy and greatly reduce the cost of manual annotation.

**Figure 10:**
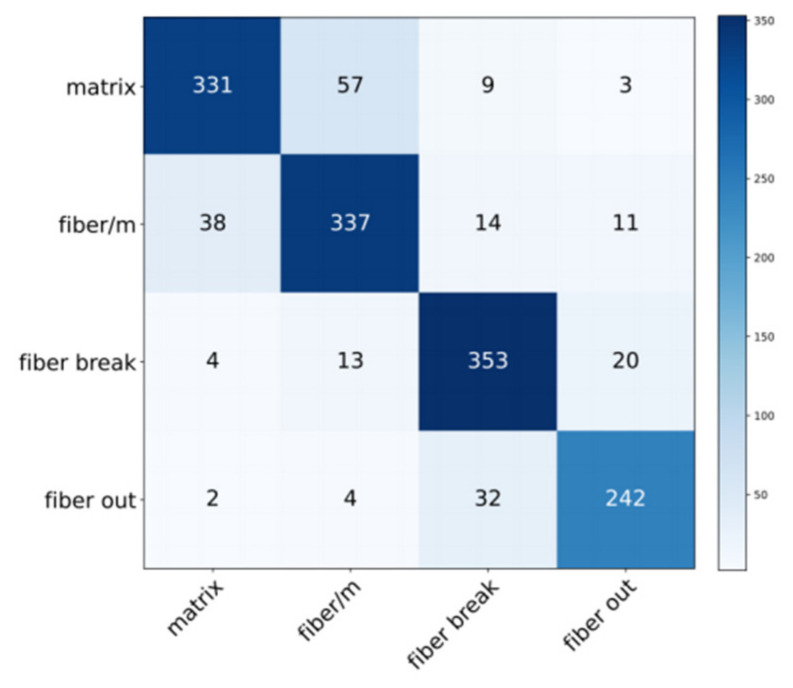
Confusion matrix.

The confusion matrix of the experimental results obtained by the model combining semi-supervised learning and transfer learning is shown in Figure 10. The data for this confusion matrix were obtained from the testing phase using the model trained on the data from Stages 1 to 7, with the labeled data from Stage 8 used for evaluation.

**Figure 11:**
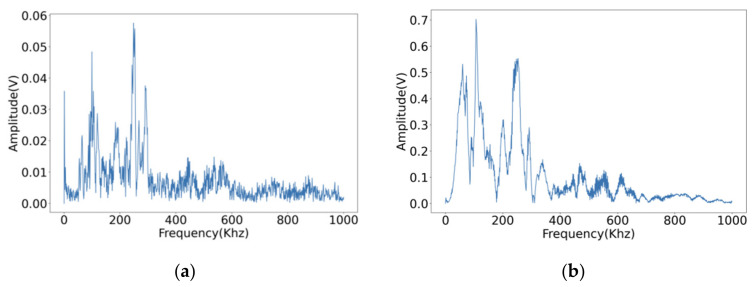
(**a**) Matrix cracking. (**b**) Fiber/matrix debonding.

It can be seen from the confusion matrix that there is more confusion between matrix cracking and fiber/matrix debonding. A deeper analysis of the confusion matrix indicates that the main classification error occurs between matrix cracking and fiber/matrix debonding. Specifically, 57 matrix cracking AE hits are misclassified as fiber/matrix debonding. This confusion is mainly caused by the strong overlap of their frequency-domain characteristics, since both damage types are concentrated in the 0–300 kHz range, as shown in Figure 11. In addition, because the labeling in this study is primarily based on peak-frequency-related characteristics, mixed or transitional signals generated during progressive damage evolution may not be completely separable. Therefore, these two damage modes exhibit similar time–frequency patterns, which makes them more difficult to distinguish from the other classes. This result indicates that the proposed model has good overall classification ability, but still shows limited discrimination for damage modes with highly overlapping spectral features. A total of 57 matrix cracking AE hits are misclassified as fiber/matrix debonding. As shown in Figure 11, it is the frequency domain diagram of fiber/matrix debonding and matrix cracking. When labeling with peak frequency, the existence of mixed signals cannot be excluded. In the figure shown below, the main frequencies of each AE hit are all distributed within 0–300 kHz, which also makes the two hit types extremely similar. Therefore, the model may produce classification errors when distinguishing between these two damage-related AE hit types. Figure 11 shows the frequency domain diagrams of fiber/matrix debonding and matrix cracking.

**Figure 12:**
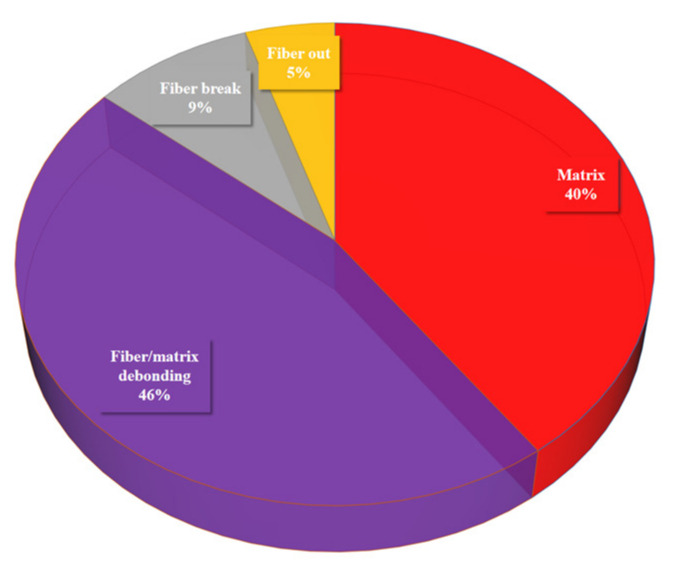
Damage distribution prediction for Stage 9.

Figure 12 presents the damage distribution prediction for Stage 9. The pie chart illustrates the proportions of different damage types, with fiber/matrix debonding (46%) accounting for the largest portion, followed by matrix cracking (40%). Fiber breakage makes up 9%, while fiber out is the smallest category at 5%. This distribution provides insights into the dominant damage mechanisms in Stage 9, with fiber/matrix debonding being the most prevalent type of damage.

## 5. Conclusions

In the current data-driven era, the acquisition of labeled data is often an expensive and time-consuming process. Therefore, how to achieve efficient and accurate model training under limited labeled data has become an important research direction. To address this issue, we propose a gas cylinder stage damage distribution prediction algorithm based on semi-supervised and transfer learning algorithms. This algorithm can still achieve effective and accurate damage classification with only a small amount of labeled data, which reduces the need for extensive manual annotation and enhances the engineering practicality of AE-based monitoring for composite gas cylinders. After conducting a staged gas cylinder pressurization experiment, the obtained burst-type AE hits were analyzed and each hit was labeled with one of the four damage types. The classification unit is thus the individual AE hit, not an aggregated event. The fully supervised and semi-supervised network structures were compared and further improved by combining transfer learning. The results show that combining transfer learning and semi-supervised learning can effectively improve the recognition result of gas cylinder damage type classification.

Furthermore, the study shows that the damage distribution prediction based on experimental data effectively reveals the dominant damage types at different stages of the gas cylinders, particularly fiber/matrix debonding and matrix cracking. These damage types occupy the majority of the distribution. The predictive model allows for an accurate assessment of the damage evolution trends, providing strong theoretical support and practical guidance for future damage monitoring and safety evaluations. These predictive results are crucial for optimizing damage detection and prediction models, ultimately enhancing the safety of gas cylinders during use.

## Figures and Tables

**Table 1:** Main technical parameters.

Main Technical Parameters	
Nominal working pressure	2.1 MPa
Hydrostatic test pressure	3.2 MPa
Water volume	18 L
Air tightness test pressure	2.1 MPa

**Table 2:** Gas cylinder layer design.

Gas Cylinder Layer Design	
Winding tension	80–150 N
Polar hole radius	23 mm
Radius of cylinder section	146 mm
Thickness of single winding layer	0.75 mm in hoop direction and 1.67 mm in longitudinal direction

**Table 3:** Acoustic emission acquisition parameter settings.

Acoustic Emission Acquisition Parameter Settings					
Pre-amplifier gain	Pre-trigger time	PDT	HDT	HLT	Sampling rate
40 dB	256 μs	200 μs	800 μs	1000 μs	1 MHz

**Table 4:** Total number of recorded AE hits and manually labeled AE hits at each stage.

Stage	Total AE Hits	Matrix Cracking	Fiber/Matrix Debonding	Fiber Fracture	Fiber Pullout
1	36	0	1	0	0
2	134	2	4	1	3
3	345	8	5	1	2
4	1033	14	16	17	14
5	3866	59	58	54	42
6	9056	127	98	109	110
7	15,355	190	217	218	219
8	19,881	400	400	390	280
9	23,426	/	/	/	/

**Table 5:** Performance comparison of different methods.

Method	Acc	Precision	Recall	F1-Score
K-means [23]	25.37 ± 0.78	18.03 ± 0.84	23.81 ± 0.73	17.53 ± 0.81
Supervised learning [24]	47.62 ± 0.69	62.19 ± 0.74	44.85 ± 0.71	42.02 ± 0.76
Supervised learning + Transfer learning	55.78 ± 0.58	64.90 ± 0.62	56.27 ± 0.60	52.26 ± 0.57
Semi-supervised learning	85.03 ± 0.41	86.03 ± 0.93	84.62 ± 0.44	85.07 ± 0.40
Semi-supervised learning + Transfer learning	85.92 ± 0.82	86.12 ± 0.26	85.98 ± 0.24	86.01 ± 0.25

Note: Results are presented as mean ± standard deviation over 10 independent runs.

## Data Availability

Data can be provided upon request from the corresponding author.
